# Peptides for Skin Protection and Healing in Amphibians

**DOI:** 10.3390/molecules24020347

**Published:** 2019-01-18

**Authors:** Ilaria Demori, Zeinab El Rashed, Viola Corradino, Annamaria Catalano, Leila Rovegno, Linda Queirolo, Sebastiano Salvidio, Emanuele Biggi, Matteo Zanotti-Russo, Laura Canesi, Alessandro Catenazzi, Elena Grasselli

**Affiliations:** 1 Dipartimento Scienze della Terra dell’Ambiente e della Vita—DISTAV, Università degli Studi di Genova—16132 Genova, Italy; idemori@unige.it (I.D.); Zeinab.ALRashed94@hotmail.com (Z.E.R.); violacorradino@outlook.it (V.C.); leila.rovegno@gmail.com (L.R.); queirolo.linda@gmail.com (L.Q.); salvidio@dipteris.unige.it (S.S.); laura.canesi@unige.it (L.C.); 2 iDelivery iSrl, Via Eremo al Santuario n.75/A, 89124 Reggio Calabria, Italy; catalano@idelivery.it; 3 International League of Conservation Photographers, Arlington, VA 22203, USA; ebiggi@anura.it; 4 Angel Consulting via San Senatore 14, 20122 Milano, Italy; info@angelconsulting.eu; 5 Department of Biological Sciences, Florida International University, Miami, FL 33199, USA; acatenazzi@gmail.com or acatenazzi@siu.edu

**Keywords:** keratinized tegument, amphibian skin peptides, wound healing, UV-irradiation protection, skin defenses, eco-physiology, animal physiology, *Batracochytrium dendrobatidis*

## Abstract

Amphibian skin is not to be considered a mere tegument; it has a multitude of functions related to respiration, osmoregulation, and thermoregulation, thus allowing the individuals to survive and thrive in the terrestrial environment. Moreover, amphibian skin secretions are enriched with several peptides, which defend the skin from environmental and pathogenic insults and exert many other biological effects. In this work, the beneficial effects of amphibian skin peptides are reviewed, in particular their role in speeding up wound healing and in protection from oxidative stress and UV irradiation. A better understanding of why some species seem to resist several environmental insults can help to limit the ongoing amphibian decline through the development of appropriate strategies, particularly against pathologies such as viral and fungal infections.

## 1. Introduction

In vertebrate evolution, a keratinized tegument appeared for the first time in amphibians, giving these animals the chance to permanently abandon the aquatic environment and become fully terrestrial, one of many changes that gave rise to the evolution of tetrapods. The amphibian skin not only represents a physical protection from the external environment, but also performs various functions such as respiration, osmoregulation, and, to a limited degree, thermoregulation [1]. During the transition from water to land, several strategies evolved to protect the skin from both endogenous and exogenous insults. Here, we review one such strategy, the secretion of skin peptides by granular glands in the dermis, focusing on the protective actions of such genome-encoded molecules, which seem able to give additional and species-specific resources for survival in changing environments.

## 2. Amphibian Skin Glands

The amphibian dermis is located beneath the epidermis and it can be divided into two different layers: the *stratum spongiosum* and *stratum compactum*, corresponding to the papillary and reticular dermis, respectively, in humans [2].

**Figure 1:**
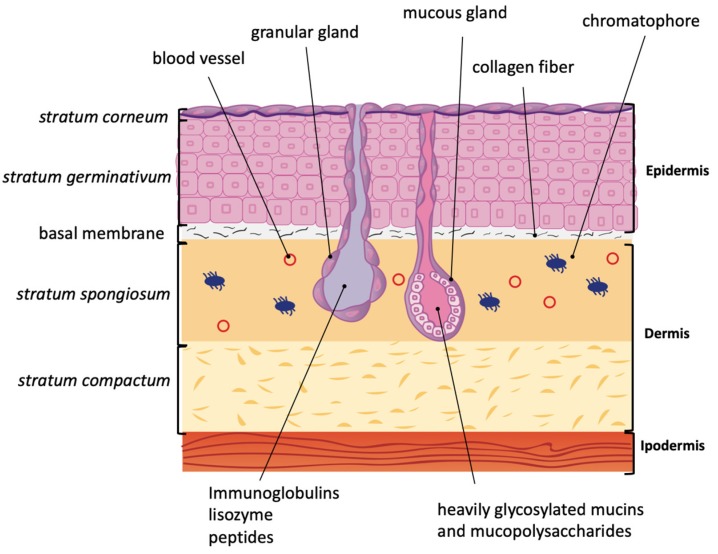
Amphibian skin anatomy. Amphibian epidermis is composed of the *stratum corneum* (only one layer of keratinized cells) followed by a regenerative basal layer, the *stratum germinativum*. These two layers are separated by irregular intracellular spaces that are interrupted by desmosomes. The *stratum germinativum* is usually 4–8 cell thick, with a progressive changing of shape from columnar to shorter from the innermost layer to the outermost. Collagen fibers reach basal membrane and separate epidermis from dermis. The latter is formed by *stratum spongiosum* and *stratum compactum*. In the *stratum spongiosum,* granular and mucous glands are present. Chromatophores, responsible of multi-colored amphibian skin, are also present in the dermis.

The amphibian dermis is constellated by a plethora of mucous and granular glands (Figure 1) [1,3]. Mucous glands produce heavily glycosylated mucins and mucopolysaccharides, which counteract water loss by maintaining skin humidity [1,4]. Poison/serous/granular glands can secrete various poisonous compounds as defense from predators. These glands are also responsible for the production of an array of proteins and peptides such as immunoglobulins, lysozymes, neuropeptides, and skin peptides [5,6,7]. A layer of myoepithelial cells equipped with adrenoreceptors surrounds the granular glands. Any kind of stress induces the release of epinephrine and/or norepinephrine, which in turn triggers the contraction of myoepithelial cells, squeezing the granular gland, and thus causing the release of gland content on the surface of the skin. These gland secretions protect the amphibian from the primary stress cause [8].

In addition to mucins, mucopolysaccharides, and peptides secreted by serous glands, the amphibian skin hosts a large variety of bacteria and bacteria-associated metabolites that together constitute the so called mucosome [9,10].

**Figure 2:**
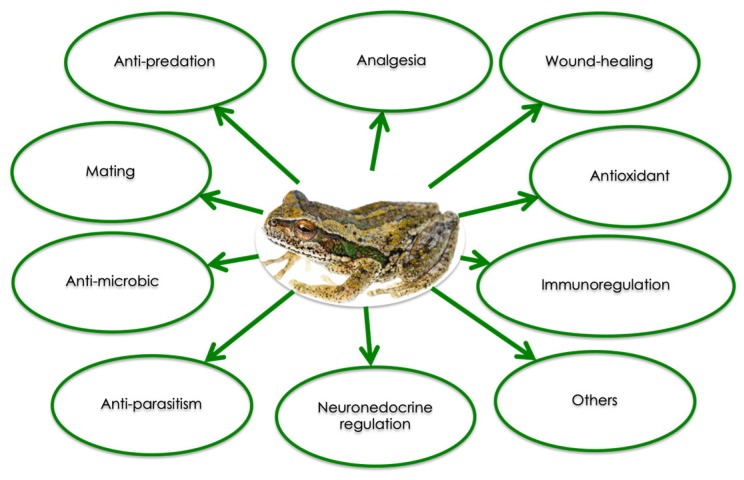
Summary of the biological activities of amphibian skin peptides (Modified from [9]).

Each amphibian species produces its own specific set of peptides with well-defined sequences. Since 2015, more than 2000 peptides from amphibian skin have been characterized [11]. Beside their anti-microbial and anti-fungal activities, skin peptides have been shown to exert other biological effects (Figure 2), and can be divided into several categories according to their function: myotropical peptides, opioid peptides, corticotropin-releasing peptides, angiotensins, protease inhibitor peptides, neuropeptides, antioxidant peptides, lectins, insulin-releasing peptides, mast cell degradation/histamine-releasing peptides, wound healing promoting peptides, immunomodulatory peptides, neuronal nitric oxide synthase inhibitors, antimicrobial peptides, antiviral peptides, antitumor peptides, antiparasite peptides, pheromone peptides, granains, and other peptides [12,13,14].

## 3. Wound Healing Process

**Figure 3:**
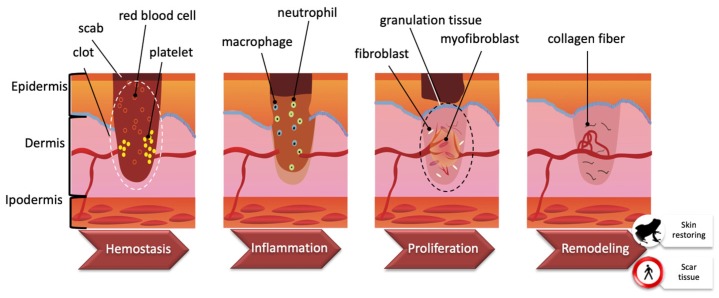
Wound healing process. Wound healing is a complex process encompassing a number of overlapping phases, including hemostasis, inflammation, proliferation, and remodeling. See the text for further explanation (Modified from [15]).

Skin has the fundamental function of separating the internal and external environments. Among all tissues, the skin can be exposed to several insults such as dehydration, ultraviolet exposure, and wounds, such that wound healing (WH) is essential for all organisms to survive [15]. WH is an evolutionarily conserved process that requires the coordination of many different cell types and cellular pathways. Generally, there are four highly integrated and overlapping phases in WH, including hemostasis, inflammation, proliferation, and tissue re-modelling leading to wound resolution [16,17]. These stages are schematized in Figure 3. Hemostasis starts immediately after a wound is produced. Platelets adhere to damaged blood vessel walls and trigger blood clotting by activating coagulation factors. Once the clot is formed, it includes platelets, red blood cells, and extracellular matrix molecules. Within a few hours/days, the inflammatory phase begins and phagocytic neutrophils and macrophages enter the clot. Chemokines secreted by macrophages are responsible for subsequent events such as angiogenesis, matrix deposition, re-epithelialization, and fibroblast migration. The formation of granulation tissue results in re-epithelialization by migration and proliferation of fibroblasts and endothelial cells. The temporary matrix is progressively collagen-enriched by fibroblasts, which then transform into myofibroblasts. Finally, the transition from granulation to scar tissue occurs [18,19].

Despite their distance in terms of evolution, amphibians and mammals share the same evolutionary ancestry, basic architecture, and conserved mechanisms underlying WH [20], so that amphibians can serve as a valuable model to study this process. Moreover, amphibians display a strong ability for high efficiency of wound repair up to the point of regenerating an entire limb or tail in juvenile animals [21,22,23] (for a detailed description, see [19]).

WH in amphibians has been extensively studied to clarify the molecular mechanisms leading to complete repair of tissue architecture and function [24,25,26]. However, this process of tissue restoration is not accompanied by scar formation, as occurs in mammals. The speed of wound closure is the most evident difference between amphibian and mammal WH. In the axolotl, a wound with a diameter of 1.5 mm can be fully re-epithelialized within 8 h [24]. In contrast, the re-epithelialization of even a small lateral wound in mammals can take between 48 and 72 h [19].

In amphibians, the injury of skin triggers hemostasis in a few seconds. No more than one day is required for the migration of epidermal cells, through pseudopodial projections, and for wound closure. Within one day, the underlying subcutaneous musculature in direct contact with the wound epidermis is disrupted [24,26]. At this site, a few mononuclear cells are recruited and reach a great number in four days. Eosin-negative, highly proliferative cells similar to mononuclear cells appear in the surrounding dermal layer. These cells seem to be the source of mesenchymal cells and express paired-type homeobox-containing transcription factor (Prx1) and T-box transcription factor 5 (Tbx5), markers of blastema cells in limb regeneration [23,24,26]. It is suggested that activation of Prx1 may be required for scarless skin WH. The mouse Prx1 enhancer possesses all elements required for its activation in WH, but such activation is not reported at skin wound sites in mice [26]. In amphibians, a nearly normal dermis and well-organized muscle begin to reappear within 10 days after injury [24,26]. Within a couple of months, the wound is indistinguishable from the surrounding skin, and no scar is formed, indicating that the WH process is complete.

## 4. Amphibian Peptides Involved in WH

Several peptides secreted by granular glands are able to promote wound closure and re-epithelization. Their WH ability has been demonstrated in several human cell types, given the scarcity of amphibian models. However, the conservation of the WH process throughout evolution allows us to speculate that the effects observed on human cells likely may also occur in amphibians.

Frogs belonging to the *Bombina* genus, such as *B. maxima* or *B. variegata*, produce the peptide bombesin (Table 1), which promotes cell proliferation and migration in different experimental models of WH. In an experimental model of mechanical injury to human keratinocytes, treatment with bombesin modulates the expression of several important skin repair factors such as TGF-β, IL-8, COX-2, VEGF, and TLR2, and promotes migration, proliferation, and neoangiogenesis in the damaged tissue. Bombesin purified from amphibian skin is homologous to mammalian gastrin-releasing peptide and is active in mammals; this similarity may justify the several biological activities exhibited by this peptide [27].

Alytesin (Table 1) is a WH peptide isolated from the amphibian *Alytes obstetricans* with pharmacological activity close to bombesin [28].

Peptides belonging to the trefoil family are characterized by having at least one copy of the trefoil motif, a 40-aminoacid sequence, in which six cysteines are in disulfide-form in 1–5, 2–4, and 3–6 configurations resulting in a three-looped structure. In mammals, trefoil factor family (TFF) proteins are involved in mucosal maintenance and repair, and they are also implicated in tumor suppression and cancer progression. One of the essential biological effects of TFFs is repair and healing of injury by stimulating the migration of cells at the mucosal wound edges. A novel two domain TFF protein named Bm-TFF2 (Table 1), isolated from *B. maxima* skin secretions, can activate human platelets in a dose-dependent manner by inducing integrin alpha(IIb)beta(3) [29]. Preliminary studies of Bm-TFF2 on toad primary skin cells also indicated that Bm-TFF2 could stimulate the toad cell migration in vivo, resulting in a faster WH process [30].

The calcitonin gene-related peptide (CGRP) from the skin of the frog *Phyllomedusa bicolor* (PbCGRP) (Table 1) is homologous to the human one (hCGRP). The hCGRP is a potent neuropeptide acting as an antagonist of the CGRP-1 receptor [31]. In humans, hCGRP has been previously described to influence proliferation of several skin cell types, such as endothelial cells, Schwann cells, and epithelial cells. The C-terminal fragment of hCGRP, hαCGRP8-37, behaves as a competitive antagonist at the CGRP-1 receptor [31]. As compared to its human counterpart, pbCGRP, which binds to the CGRP-1 receptor, has a higher antagonistic potency and affinity, especially due to its C-terminal fragments. Thus, pbCGRP can exert WH properties through binding to CGRP-1 receptors.

The 2.6 kDa peptide called AH90 (Table 1) isolated from *Odorrana grahami* shows a strong wound closure capacity in both in vitro and in vivo WH models. AH90 powerfully promotes in vitro migration of human keratinocytes (HaCaT), thus indicating a promotion of superficial wound closure. Even topical application of AH90 on Balb/c mice significantly accelerated wound closure through dermal regeneration, granulation tissue formation, and deformation, resulting in a thinner epidermal thickness. The hypothesis is that AH90 might induce granulation tissue contraction by promoting transition of fibroblasts to myofibroblasts, which is a key step in both the WH process and matrix remodelling. AH90 also increases cell adhesion to fibronectin and laminin, which is likely promoted by an up-regulation of five and six integrins. AH90 treatment for 7 days greatly increased the levels of smooth muscle actin (SMA), the marker of myofibroblast differentiation, at the wound border, throughout the granulation tissue and in blood vessel walls. AH90 could induce transforming growth factor β1 (TGF-β1) secretion through activation of the JNK and NF-κB signaling pathway. The secretion of TGF-β1 could cause an increase in SMA expression through phosphorylation of Smad3 and play a major role in myofibroblast differentiation. Moreover, WH has been demonstrated to speed up in mice models by stimulating TGF-β1 secretion [32].

Temporins A and B (Table 1) are two WH peptides, isolated from *Rana temporaria,* whose proliferative ability has been demonstrated in vitro by using HaCaT cells. Both temporin A and B can directly or indirectly stimulate cell surface epidermal growth factor receptor (EGFR) and activate downstream signaling cascades. Temporins induce migration of HaCaT keratinocytes, which appear to be mediated by EGFR. These peptides are also effective in fighting skin infections, such as those provoked by *Staphylococcus aureus*, that could slow down wound closure [33].

The OA-GL21 (Table 1) peptide isolated from by *Odorrana andersonii* exerts the ability to promote the WH of human keratinocytes and human fibroblasts in a dose- and time-dependent manner, mainly by promoting cell migration rather than inducing cell proliferation [34].

Cathelicidin-OA1, secreted by *O. andersonii*, (Table 1) belongs to a group of cationic peptides (Cathelicidins) with amphipathic properties. These peptides usually are active in inhibiting bacterial growth. On the other hand, Cathelicidin-OA1 does not exert antimicrobial activity, but is the first cathelicidin identified from an amphibian that shows potent WH ability both in vivo and in vitro. In cellular models, this peptide promotes both HaCaT cell proliferation and human skin fibroblast (HSF) cell migration. In a mouse model with deep skin wounds, Cathelicidin-OA1 accelerates re-epithelialization and granulation tissue formation by enhancing the recruitment of macrophages to the wound site, with effects comparable to those exerted by epidermal growth factor (EGF). TNF-α, a pro-inflammatory cytokine, can be upregulated by peptides in the early phase of inflammation during WH, thus becoming responsible for recruiting inflammatory cells to the wound site and exerting predominant chemotactic functions [34].

OM-LV20 (Table 1), from *Odorrana margaretae*, has demonstrated significant WH potency both in vivo and in vitro. Topical skin application of OM-LV20 on skin wounds in mice significantly accelerates healing. OM-LV20-induced wound healing in HaCaT cells is related, but not limited to, cell proliferation. Finally, OM-LV20 stimulates human skin fibroblast (HSF) WH via cell migration, cell adhesion, or fibroblast to myofibroblast transition, rather than by proliferative effects [35].

CW49 (Table 1) isolated from *Odorrana grahami* has a strong pro-angiogenic ability in wounds of both normal and diabetic mice. Angiogenesis is a key process in normal WH. In diabetic wounds, angiogenesis is often impaired, thus strongly limiting wound closure. Furthermore, CW49 significantly reduces the number of infiltrated macrophages in wounds. This effect might not be good for the WH process in normal wounds, although it might be beneficial for WH in diabetes by preventing the excessive inflammatory response typical of diabetic wounds [36].

Experiments performed utilizing isolated amphibian skin peptides allow us to understand the effects and possibly the mechanisms of action of a molecule without any interference from other molecules. However, amphibians secrete a mixture of molecules, including other peptides and metabolites, some of which are also produced by symbiotic skin bacteria. Moreover, it should be considered that the effects of different substances contained in a mixture can be additive, synergistic, and antagonistic. Thus, from an eco-physiological perspective, the effects of peptide mixtures on the WH process can be more representative of the actual mechanisms occurring on the amphibian skin in the wild.

Acid and pepsin-solubilized peptide mixtures (ARP and ERP) from *Rana chensinensis* contain peptides ranging from 190 to 500 Da. ARP and ERP exhibit significant proliferative and anti-apoptotic effects on HaCaT keratinocytes. ERP has a greater bioactivity than ARP at equal concentrations. Differences in molecular weight and amino acid composition may explain variation in bioactivity between ARP and ERP. EGF could be a mediator of peptide mixture actions by regulating the survival and proliferation of cells through several downstream pathways (AKT, ERK, STAT3, and JNK). The anti-apoptotic effect exerted by both ARP and ERP, with ERP being more effective, is mediated by a reduction of expression of intracellular caspase-3 protein [37].

WH properties of peptide mixtures isolated from *Gastrotheca excubitor* and *G. nebulanastes* have been analyzed in our lab in order to investigate the possible mechanisms underlying the susceptibility of amphibians to *Batrachochytrium dendrobatidis* (*Bd* [38]). *Bd* is a pathogenic chytrid fungus that invades the amphibian skin with motile zoospores, which encyst and proliferate in keratinized dermal tissues [39,40], thus causing the onset of chytridiomycosis, loss of the integument functionality, skin ulcers, and eventually animal death [41,42]. *Bd* is responsible for the extinction of entire genera of amphibians, thus causing pauperization of amphibian biodiversity. It is noteworthy that *Bd* susceptibility is different depending on species-specific mechanisms not yet fully understood. As an example, *G. excubitor* and *G. nebulanastes*, despite belonging to the same genus, display different susceptibility to *Bd* infection, with *G. excubitor* being much more resistant. However, we failed to demonstrate healing properties for peptide mixtures from *G. excubitor* and *G. nebulanastes* in an in vitro cellular model of WH (see Supplementary Materials and Figure S1 for details about the experiments). Nevertheless, Burkart et al. [43] demonstrated that cutaneous bacteria diversity between the two species was responsible for different degrees of *Bd* growth inhibition effects and it has been shown that skin secretions from other frog species strongly inhibit *Bd* growth in vitro, thus contributing to species resistance to chytridiomycosis [44,45,46,47,48]. All these observations confirm that defense mechanisms of amphibian skin are complex and species-specific, involving different pathways that still need to be elucidated.

## 5. Amphibian Peptides Known for Antioxidant/Free Radical Scavenging Activities

Reactive oxygen species (ROS) include superoxide anions, peroxides, hydroxyl radicals, and singlet oxygen. ROS are able to damage virtually all categories of biological molecules: proteins, DNA, RNA, and lipids. This damage can trigger several harmful cellular processes, ultimately leading to cell death. The skin is subjected to both endogenous and exogenous insults, most of them leading to ROS production [50]. ROS are mainly generated by keratinocytes in response to signals from cytokines, growth factors, airborne pollutants, UV radiation, and physiological stimuli [51].

Given the complexity of amphibian skin function, the need to maintain skin integrity in both aquatic and terrestrial environments, and the fragility of the *stratum corneum,* it is imperative for amphibians, more than for other vertebrates, to efficiently protect the skin against oxidative stress. Amphibians have developed a multifaceted and effective system composed of antioxidant enzymes and small molecules that can act as ROS scavengers [51].

Among non-enzymatic antioxidants, skin peptides are the most represented category (Table 2). Their antioxidant activity is related to their aminoacidic composition, arrangement, and secondary structures. The presence of reductive cysteine in the primary structure strongly supports their antioxidant activity, because the reductive mercapto group (-SH) in cysteine confers a stronger antioxidant activity with respect to other amino acids such as tyrosine, tryptophan, methionine, or proline [52].

Pleurain, isolated from *Rana pleuraden,* is a family of 10 members all with antioxidant activity, but some of them also show anti-microbial and anti-inflammatory properties (Table 2). Interestingly, all the antioxidant pleurains have similar precursor structures. Furthermore, their precursors are similar to skin peptide precursors of ranid amphibians. They share a similar overall structure that is composed of an *N*-terminal peptide followed by an acidic spacer peptide and a *C*-terminal mature peptide. These antioxidant peptides contain proline residues, suggesting that proline may also have an important antioxidant activity. Among the pleurain family, pleurain-*E*, pleurain-*N*1, and pleurain-R1 have been demonstrated to exert the strongest free radical scavenging ability [53].

Antioxidin-RP1 and RP2 are mainly antioxidant and weakly anti-inflammatory (Table 2). Both contain proline residues. Antioxidin-RP1 has been found to be the most potent antioxidant peptide secreted by *Rana pleuraden* and to contain most of the aminoacids responsible for antioxidant function. In addition to Cys10, antioxidin-RP1 also possesses two tyrosine residues (Tyr6 and Tyr12) with different antioxidant capabilities, suggesting that not only the chemical nature of residues but also their position confer different antioxidant power to the peptide [9,53].

The activities of amphibian skin-derived peptides are often overlapping, thus, it is of note that peptides indicated to exert a strong WH power also present weak antioxidant activities, for example, OA-GL21 [34], OM-LV20 [35], and Cathelicidin-OA1 [49] (Table 2).

## 6. Amphibian Peptide Production for Ultraviolet Irradiation Adaptation

Skin peptides can serve as molecular mechanisms for fighting the harmful effects of UV. There are three types of ultraviolet radiation: UV-A (315–400nm), UV-B (280–315 nm), and UV-C (200–280 nm). Long-waved UV-A radiation is more than 95% of the total UV radiation reaching the Earth’s surface [55]. UV-A radiation can lead to the production of ROS, thus triggering skin and other tissue damage [56]. UV-B radiation has increased over the last few decades due to stratospheric ozone depletion. UV-B radiation can kill amphibian embryos and/or cause sublethal effects that can harm amphibians in later life stages [57]. Strategies to counteract the side-effects of UV radiation include behavioral, physiological, and molecular defenses. Thus, the marked difference in skin peptide composition and complexity that can be observed even in species from the same genus, might reflect adaptation to different environments characterized by different sunlight exposure.

An excellent example comes from the paper of Yang et al. [58] comparing *Odorrana andersonii* with *O. wuchuanensis*. *Odorrana andersonii* lives at 2500 m altitude, in an environment characterized by exposure to sunshine and strong UV radiation, while *O. wuchuanensis* is distributed in a small number of caves (altitude 800 m) and is not exposed to sunlight during its entire life cycle (Table 3). Exposure to UV radiation resulted in different composition of skin secretion in both amphibians. With respect to *O. andersonii*, UV-irradiated skin of *O. wuchuanensis* showed tissue damage, indicating a difference in the amount and aminoacidic composition of secretions between the two species. Skin secretions from *O. andersonii* demonstrated more antioxidant ability than those from *O. wuchuanensis:* 42 vs. 5 peptides with antioxidant properties were isolated from the two species, respectively. Among the peptides isolated from *O. andersonii* and *O. wuchuanensis,* Andersonin-AOP1 and Wuchuanin-AOP5 were the most potent antioxidants, with Andersonin-AOP1 being 20-fold more powerful than Wuchuanin-AOP5. Yang et al. demonstrated that *O. andersonii* produces and secretes a more complex mixture of peptides with a more powerful antioxidant capacity, thus increasing fitness in a hostile environment characterized by high exposure to UV radiations (Table 3).

On the other hand, the production of antioxidant peptides may be related to long-term exposure to strong UV light in an oxygen-rich atmosphere and to behaviors related to weather [14,53]. The comparison of the four frog species *Amolops daiyunensis, Pelophylax hubeiensis, Hylarana maosuoensis,* and *Nanorana pleskei* showed that the weakest antioxidant power can be attributed to peptides isolated from the frog living in the highest environment (*N. pleskei* at 3800 m)*,* whereas peptides isolated from *P. hubeiensis*, living at 50 m altitude, presented the strongest antioxidant activity. This can be due to a behavioral component related to the environment. Given the extreme altitude of its habitat, *N. pleskei* is exposed to sunlight only for a short period of time during summer, whereas *P. hubeiensis* can be observed on lotus leaves all through the year, because warmer temperatures allow frogs to be active throughout the year and experience longer sunlight exposure [54] (Table 3).

## 7. Conclusions

Amphibians are currently suffering a dramatic decline worldwide, and the question “Why are some species in decline but others are not?” is intriguing ecologists, conservation biologists, wildlife managers [61], and even the informed layman [62]. Different aspects of amphibian ecology, ecophysiology, and behavior have been considered to explain the observed patterns of decline such as phylogeny, geographic distribution, microhabitat selection, patterns of activity (i.e., nocturnal vs. diurnal), and other life history characteristics. However, a clear and univocal explanation remains elusive [63]. In any case, it is clear that some species are able to tolerate human-mediated environmental changes observed both at the local (e.g., land-use transformation, pollutants) and at the global level (e.g., climate change) better than others. These species might acquire tolerance to unpredictable environmental alterations by possessing a more complex array of innate behavioral, ecophysiological, or immunological features that are still poorly understood [64,65].

As first lines of defense for amphibians to survive environmental challenges, skin protection and healing are essential, since in these vertebrates the cutis performs a multitude of functions related to respiration, osmoregulation, and thermoregulation. Amphibian skin peptides play a major role in maintaining skin integrity and functionality, which is important in allowing these animals to resist continuous physical, chemical, and biological threats. On the other hand, although outside the focus of our review, the preliminary results on WH on human cell lines using amphibian peptides clearly show the huge potential of these molecules for the benefit of human health and, therefore, this issue needs further consideration and more in-depth studies [33,66].

Although the mechanisms underlying the protective effects of skin peptides are far from being elucidated, it is of note that most of these molecules exhibit anti-oxidant properties related in particular to the presence in the peptide primary structure of the reductive mercapto group (–SH) in cysteine. It is well known that oxidative stress is linked to inflammation since overproduction of mitochondrial ROS promotes the synthesis of pro-inflammatory cytokines [67], thus suggesting that precise cysteine positions can also trigger other cellular events than mere oxidative stress protection. It is conceivable that anti-oxidant and anti-inflammatory actions of skin peptides are mainly involved in the defense mechanisms that protect the skin of amphibians living in environments characterized by strong UV radiation, which trigger ROS production in keratinocytes. However, anti-oxidant skin peptides may well contribute to speeding up the WH process, which is highly efficient in amphibians and entails an inflammatory phase with the involvement of immune cells and cytokines. Of note, all peptides belonging to the trefoil family, characterized by six cysteines in disulfide-form, show WH promoting properties [29,30].

In this regard, WH and anti-oxidant abilities can be important to limit and heal skin ulcers like those due to fungal diseases, such as chytridiomycosis, which has caused the greatest disease-driven loss of biodiversity reported by science, with documented mass die-offs, community collapse, and the extinction of possibly hundreds of species [68]. Fungal infections are dangerous to all organisms because there are so few effective drugs to treat them. In amphibians, the skin provides a first line of defense against infections, and its mucous contains a rich pharmacopeia of compounds providing resistance against microbial infections. Skin peptides are likely to play an important role in regulating host-pathogen interactions, and may provide insights into understanding past and ongoing amphibian declines, as well as opportunities to develop ex-situ treatment strategies for fungal infections in amphibians and other vertebrates with the aim of preserving species even if in captive conditions.

In this framework, the synergic action of skin peptides and bacteria hosted in the skin in the mucosome could also play a relevant role in increasing resistance of amphibians to injuries caused by UV radiation and emerging infectious diseases, such as ranaviruses and chytridiomycoses. In fact, some aspects of the amphibian skin microbiome are beginning to be investigated with success [69], but the interactions of peptides and bacterial metabolites remain to be fully understood.

Besides genome-encoded peptides, other molecules are able to exert beneficial effects on amphibian skin. Among organic compounds, trimethylamine *N*-oxide (TMAO) is found in amphibian skin and acts as an osmolyte together with other inorganic osmolytes [70]. TMAO can also serve as an antioxidant, thus contributing to the maintenance of oxidative homeostasis together with antioxidant peptides [71]. On the other hand, the inorganic compound hydrogen sulfide is present in amphibians’ blood stream [72], and it can have beneficial effects on the cardiovascular system by promoting angiogenesis, thus ultimately favoring the WH process [73].

Therefore, the complex interplay among different pathways of the entire mucosome activated in skin defense of amphibians and the species-specificity of defense mechanisms need to be further elucidated in order to better understand amphibian declines and to prevent any further loss of wildlife and biodiversity.

## Figures and Tables

**Table 1:** Wound healing peptides relevant for amphibian skin defense. Secondary structure prediction was performed by GOR IV algorithm (https://npsa-prabi.ibcp.fr/cgi-bin/npsa_automat.pl?page=/NPSA/npsa_gor4.html); Hh: Alpha helix; Ee: Extended strand; Cc: Random coil.

Peptide Name	AA Sequences	Species	Length (AA)	Secondary Structure Prediction	Ref.
**AH-90**	ATAWDFGPHGLLPIRPIRIRPLCG	*Odorrana grahami*	24	Ee: 29.17% Cc 70.83%	[32]
**Alytesin**	Pyr-GRLGTQWAVGHLM	*Alytes obstetricans*	14	Ee: 46.15% Cc: 53.85%	[28]
**ARPs/ERPs**	-	*Rana chensinensis*	2–20	-	[37]
**Bombesin**	Pyr-QRLGNQWAVGHLM	*Bombina bombina* *Bombina variegata* *Bombina orientalis*	14	Ee: 38.46% Cc: 61.54%	[27]
**Bm-TFF2**	GFPIYEIDNRPGCYVDPAERVAC AGAGVTKAECKAKGCCFISARR NTIWCFKLKESADAWKCAVPM NTRVACAGAGVTPAECKGKGC CFNSSYYGTVWCFKPQE	*Bombina maxima*	104	Ee: 39.42% Cc: 60.58%	[29,30]
**Cathelicidin-OA1**	IGRDPTWSHLAASCLKCIFDDLPKTHN	*Odorrana andersonii*	27	Hh: 29.63% Ee: 7.41% Cc: 62.96%	[49]
**CW49**	APFRMGICTTN	*Odorrana grahami*	11	Ee: 45.45% Cc: 54.55%	[36]
**OA-GL21**	GLLSGHYGRVVSTQSGHYGRG	*Odorrana andersonii*	21	Ee: 52.38% Cc: 47.62%	[34]
**OM-LV20**	LVGKLLKGAVGDVCGLLPIC	*Odorrana margaretae*	20	Ee: 45.00% Cc: 55.00%	[35]
**pbCGRP**	SCDTSTCATQRLADFLSRSGGIGSPDFVPTDVSANSF	*Pyllomedusa bicolor*	37	Hh: 18.92% Ee: 13.51% Cc: 67.57%	[9,31]
**Temporins-A**	FLPLIGRVLSGIL	*Rana temporaria*	13	Ee: 69.23% Cc: 30.77%	[33]
**Temporins-B**	LLPIVGNLLKSLL	*Rana temporaria*	13	Ee: 30.77% Cc: 69.23%	[33]

**Table 2:** Antioxidant peptides relevant for amphibian skin defense. Secondary structure prediction was performed by the GOR IV algorithm (https://npsa-prabi.ibcp.fr/cgi bin/npsa_automat.pl?page=/NPSA/npsa_gor4.html); Hh: Alpha helix; Ee: Extended strand; Cc: Random coil.

Peptide	AA Sequences	Species	Length (AA)	Secondary Structure Prediction	Ref.
**Andersonin-C1**	TSRCIFYRRKKCS	*Odorrana margaratae*	13	Ee: 53.85% Cc: 46.15%	[9]
**Andersonin-G1**	KEKLKLKAKAPKCYNDKLACT	*Odorrana andersonii*	21	Ee: 23.81% Cc: 76.19%	[9]
**Andersonin-H3**	VAIYGRDDRSDVCRQVQHNWLVCDTY	*Odorrana margaratae*	26	Ee: 42.31% Cc: 57.69%	[9]
**Andersonin-N1**	ENMFNIKSSVESDSFWG	*Odorrana margaratae*	17	Ee: 52.94% Cc: 47.06%	[9]
**Andersonin-R1**	ENAEEDEVLMENLFCSYIVGSADSFWT	*Odorrana margaratae*	27	Hh: 18.52% Ee: 33.33% Cc: 48.15%	[9]
**Antioxidin-RP1**	AMRLTYNKPCLYGT	*Rana pleuraden*	14	Ee: 28.57% Cc: 71.43%	[9,53]
**Antioxidin-RP2**	SMRLTYNKPCLYGT	*Rana pleuraden*	14	Ee: 28.57% Cc: 71.43%	[9,53]
**APBMH**	LEQQVDDLEGSLEQEKK	*Rana catesbeiana*	17	Hh: 35.29% Ee: 11.76% Cc: 52.94%	[9]
**APBSP**	LEELEEELEGCE	*Rana catesbeiana*	12	Ee: 16.67% Cc: 83.33%	[9]
**Hejiangin-A1**	RFIYMKGFGKPRFGKR	*Odorrana hejiangensis*	16	Ee: 31.25% Cc: 68.75%	[9]
**Hejiangin-E1**	SADQTGMNKAALSPIRFISKSV	*Odorrana hejiangensis*	22	Ee: 28.57% Cc: 71.43%	[9]
**Hejiangin-F1**	IPWKLPATFRPVERPFSKPFCRKD	*Odorrana hejiangensis*	24	Ee: 16.67% Cc: 83.33%	[9]
**Japonicin-1Npa**	FLLFPLMCKIQGKC	*Nanorana parkeri*	14	Ee: 35.71% Cc: 64.29%	[9]
**Japonicin-1Npb**	FVLPLVMCKILRKC	*Nanorana parkeri*	14	Ee: 50.00% Cc: 50.00%	[9]
**Lividin-D1**	KNNFCQVLYVWLLRLGKQCFVKFSKDVET	*Odorrana livida*	29	Ee: 51.72% Cc: 48.28%	[9]
**Macrotympanain A1**	FLPGLECVW	*Odorrana macrotympana*	9	Ee: 33.33% Cc: 66.67%	[9]
**Margaratain-A1**	VTPPWARIYYGCAKA	*Odorrana margaratae*	15	Ee: 33.33% Cc: 66.67%	[9]
**Margaratain-B1**	FFSTSCRSGC	*Odorrana margaratae*	10	Ee: 60.00% Cc: 40.00%	[9]
**Margaratain-C1**	GMLKWKNDFFHFLQWLLISCQNYFVK	*Odorrana margaratae*	26	Ee: 50.00% Cc: 50.00%	[9]
**Nigroain-B-MS1**	CVVSSGWKWNYKIRCKLTGNC	*Hylarana maosuoensis*	21	Ee: 47.62% Cc: 52.38%	[54]
**Odorranian-A-OA11**	VVKCSYRQGSPDSR	*Odorrana margaratae*	14	Ee: 42.86% Cc: 57.14%	[9]
**Odorranian-A-OA12**	VVKFSYRKGSPAPQKN	*Odorrana margaratae*	16	Ee: 37.50% Cc:62.50%	[9]
**Parkerin**	GWANTLKNVAGGLCKITGAA	*Nanorana parkeri*	20	Ee: 45.00% Cc: 55.00%	[9]
**Pleurain-A1**	SIITMTKEAKLPQLWKQIACRLYNTC	*Rana pleuraden*	26	Hh: 19.23% Ee: 30.77% Cc: 50.00%	[53]
**Pleurain-D1**	FLSGILKLAFKIPSVLCAVLKNC	*Rana pleuraden*	23	Ee: 47.83% Cc: 52.17%	[53]
**Pleurain-E1**	AKAWGIPPHVIPQIVPVRIRPLCGNV	*Rana pleuraden*	26	Ee: 30.77% Cc:69.23%	[53]
**Pleurain-G1**	GFWDSVKEGLKNAAVTILNKIKCKISECPPA	*Rana pleuraden*	31	Hh: 45.16% Cc: 54.84%	[53]
**Pleurain-J1**	FIPGLRRLFATVVPTVVCAINKLPPG	*Rana pleuraden*	26	Ee: 34.62% Cc: 65.38%	[53]
**Pleurain-K1**	DDPDKGMLKWKNDFFQEF	*Rana pleuraden*	18	E: 22.22% Cc: 77.78%	[53]
**Pleurain-M1**	GLLDSVKEGLKKVAGQLLDTLKCKISGCTPA	*Rana pleuraden*	31	Hh: 38.71% Ee: 19.35% Cc: 41.94%	[53]
**Pleurain-N1**	GFFDRIKALTKNVTLELLNTITCKLPVTPP	*Rana pleuraden*	30	Hh: 40.00% Ee: 6.67% Cc: 53.33%	[53]
**Pleurain-P1**	SFGAKNAVKNGLQKLRNQCQANNYQGPFCDIFKKNP	*Rana pleuraden*	36	Hh: 33.33% Ee: 19.44% Cc: 47.22%	[53]
**Pleurain-R1**	CVHWMTNTARTACIAP	*Rana pleuraden*	16	Ee: 37.50% Cc: 62.50%	[53]
**Schmackerin-C1**	AAPRGGKGFFCKLFKDC	*Odorrana schmackeri*	17	Ee: 35.29% Cc: 64.71%	[9]
**Tiannanin-A1**	LLPPWLRPRNG	*Odorrana tiannanensis*	11	Ee: 36.36% Cc: 63.64%	[9]
**Wuchuanin-A1**	APDRPRKFCGILG	*Odorrana wuchuanensis*	13	Ee: 38.46% Cc: 61.54%	[9]
**Wuchuanin-C1**	VFLGNIVSMGKKI	*Odorrana wuchuanensis*	13	Ee: 53.85% Cc: 46.15%	[9]
**Wuchuanin-D1**	DAAVEPELYHWGKVWLPN	*Odorrana wuchuanensis*	18	Ee: 27.78% Cc: 72.22%	[9]
**Wuchuanin-E1**	CVDIGFSPTGKRPPFCPYPG	*Odorrana wuchuanensis*	20	Ee: 10.00% Cc: 90.00%	[9]

**Table 3:** Amphibian peptides relevant for skin protection from UV light. Secondary structure prediction was performed by the GOR IV algorithm (https://npsa-prabi.ibcp.fr/cgi-bin/npsa_automat.pl?page=/NPSA/npsa_gor4.html); Ee: Extended strand; Cc: Random coil.

Peptide	AA Sequences	Species	Length (AA)	Secondary Structure Prediction	Ref.
**Andersonin-AOP 1**	FLPGLECVM	*Odorrana andersonii*	9	Ee: 44.44% Cc: 55.56%	[58]
**Antioxidin-RL**	AMRLTYNRPCIYAT	*Odorrana livida*	14	Ee: 28.57% Cc: 71.43%	[59,60]
**Daiyunin-1**	CGYKYGCMVKVDR	*Amolops daiyunensis*	13	Ee: 30.77% Cc: 69.23%	[59]
**Maosonensis-1MS1**	QYRPGSFGPLNQK	*Hylarana maosuoensis*	13	Ee: 23.08% Cc: 76.92%	[54]
**Odorranaopin-MS2**	DYSIRTRLHQESSRNVF	*Hylarana maosuoensis*	17	Ee: 52.94% Cc: 47.06%	[54]
**Pleskein-2**	FFLLPIPNDVKCKVLGICKS	*Nanorana pleskei*	20	Ee: 35.00% Cc: 65.00%	[54]
**Ranacyclin-HB1**	GAPKGCWTKSYPPQPCFGKK	*Pelophylax hubeiensis*	20	Ee: 15.00% Cc: 85.00%	[9]
**Wuchuanin-AOP 5**	TVWGFRPSKPPSGYR	*Odorrana wuchuanensi*	15	Ee: 20.00% Cc: 80.00%	[58]

## Supplementary Materials

The following are available online. Reference [74,75] are cited in the supplementary materials.
