# Supplementary material for: Peptides for Skin Protection and Healing in Amphibians
Source: Molecules. 2019 Jan 18;24(2):347. doi: 10.3390/molecules24020347 (PMC6359409; doi:10.3390/molecules24020347)
Supplement: Supplementary file 1 [file molecules-24-00347-s001.pdf]

Materials and methods

Chemicals

Chemicals were supplied by Sigma-Aldrich Corp. (Milan, Italy), unless otherwise indicated.

Peptide mixture sampling

Peptide secretion was stimulated by injecting 40 nmol norepinephrine per g body weight into the dorsal lymph sacks of *G. excubitor, G. nebulanastes*. Frogs were then placed in plastic bags with 24 mL of HPLC water to collect skin secretion. After 15 min, the frogs were removed, 1 mL of 50% HCl was added and samples were partially filtered through Sep Pak C-18 cartridges (Waters Corporation, Milford, MA, USA). Skin peptides were eluted from cartridges with a 70% acetonitrile and 0.1% HCl solution and vacuum centrifuged to dryness. The mixtures were resuspended in PBS and skin peptide concentrations were measured with a Pierce microBCA protein assay kit (Thermo Scientific, Rockford, IL, USA) using bradykinin as standard. Stock solutions at 1 mg/mL were kept at -20°C until used.

Cell culture and treatments

HECV cells (Cell Bank and Culture-GMP-IST-Genoa, Italy) are a human endothelial cell line isolated from umbilical vein. They were grown at 37 °C in Dulbecco's modified Eagle's medium High Glucose (D-MEM) supplemented with L-Glutamine and 10% FCS. For treatments, cells were grown until 80% confluence, then incubated in serum-free medium with 0.25% bovine serum albumin (BSA). Skin peptide mixture stock solutions (1 mg/mL in PBS) were serially diluted with serum-free culture medium and used for cell treatments at final concentrations of 0.005, 0.05, 0.5, 5 and 50 μg/mL.

Cell viability

The viability of HECV cells upon exposure to peptide mixtures was determined by the MTT [3-(4,5-dimethylthiazol-2-yl)-2,5-diphenyltetrazolium bromide] assay on 96-well plates. The cells were plated at a density of 15˙000 cells/well and allowed to adhere overnight. Then the skin peptide mixtures were added in serum-free medium at concentrations ranging from 0.005 to 50 μg/mL for 24 h. The MTT substrate was prepared in PBS/EDTA, added to cells in culture at a final concentration of 0.5mg/ml and incubated for 3 h. Viable cells with active metabolism converted MTT into a purple colored formazan product whose absorbance was measured at 570 nm using a plate reading spectrophotometer. The results of these experiments are shown in figure S1.

Wound healing assay

80˙000 cells/well were seeded onto 12-well plates and growed for 24 h or until confluence was reached. Then the cell monolayer was scraped with a p100 yellow pipet tip to create a ''scratch'' (Eppendorf AG, Hamburg, Germany).

After washing, scratch images were recorded under the microscope at time 0 (T0). Then the medium was replaced with fresh serum-free medium containing different concentrations of skin peptide mixtures. After 24 h, scratch images were acquired again (T24). All images were analysed using ImageJ free software (http://imagej.nih.gov/ij/) and the distances between wound edges were measured. A decrease in edge distance after 24 hours as compared to T0 indicated the migration of cells to close the wound [74].

Statistical analysis

Data are expressed as mean ± SD of at least three independent experiments performed in triplicate. Statistical analysis was performed using ANOVA with Tukey's post-test (GraphPad Software, Inc., San Diego, CA, USA).

Secondary Structure Prediction

The percentages of the main secondary structures occurring in all amphibian peptides were predicted by theoretical calculations using the GOR IV algorithm [75]. This algorithm is the fourth version of a secondary structure prediction method based on the information theory. GOR IV allowed us to establish the structural code relating the amino acid sequence and the secondary structure of a protein. This database uses as data source a pool of 267 protein structures having a resolution better than 2.5 A˚ and with a length greater than 50 residues.

74. Vergani, L.; Vecchione, G.; Baldini, F.; Grasselli, E.; Voci, A.; Portincasa, P.; Ferrari, P. F.; Aliakbarian, B.; Casazza, A. A.; Perego, P. Polyphenolic extract attenuates fatty acid-induced steatosis and oxidative stress in hepatic and endothelial cells. *Eur. J. Nutr.* **2018**, *57*, 1793-1805.
75. Garnier, J.; Gibrat, J. F.; Robson, B., GOR method for predicting protein secondary structure from amino acid sequence. *Methods Enzymol.* **1996**, *266*, 540-53.

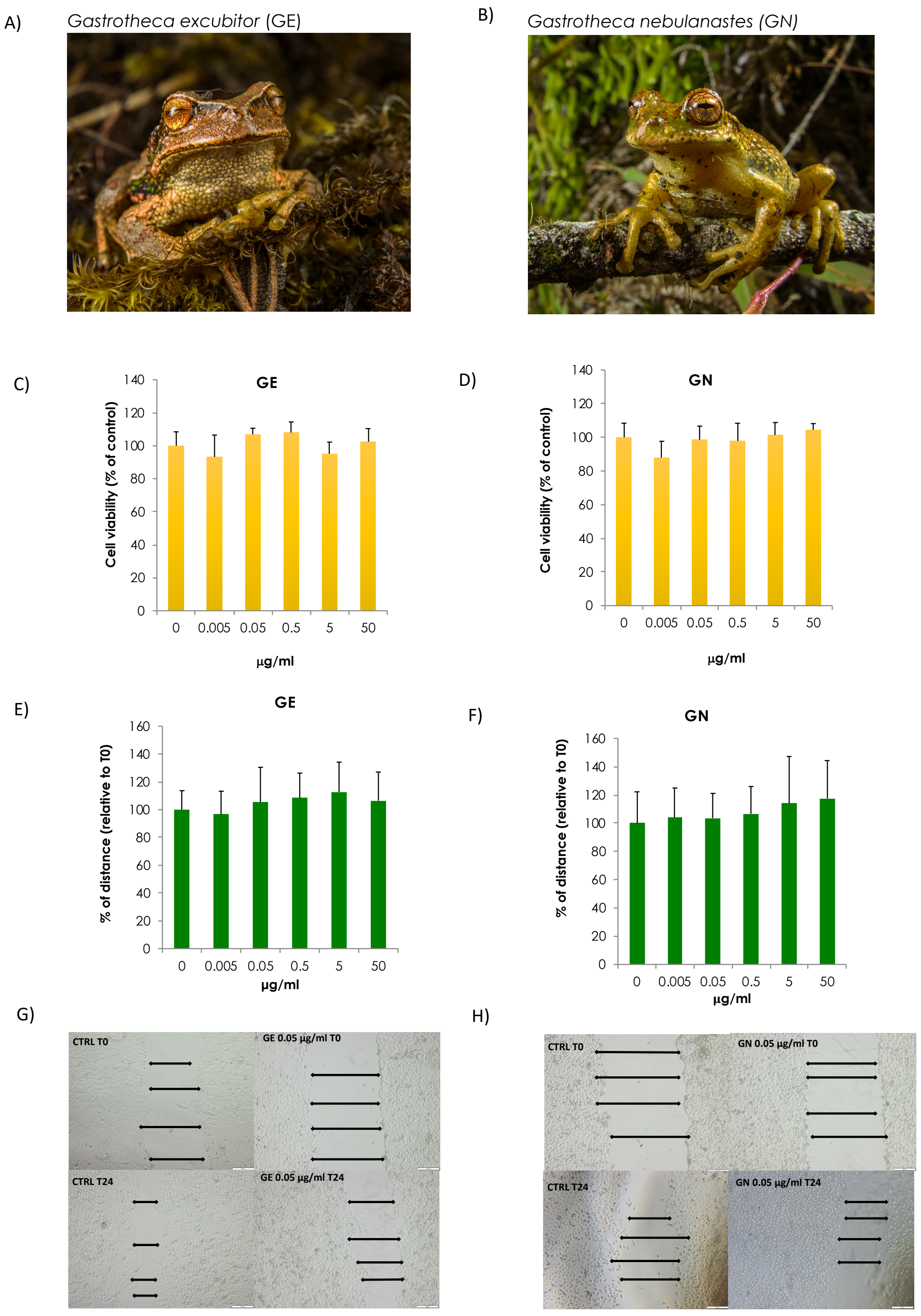

Figure S1. Effects of skin peptide mixtures on wound healing. Effects of skin peptide mixtures from *G. excubitor* (GE, A) and *G. nebulanastes* (GN, B) on cell viability (C and D respectively) and WH process (E and F respectively) in the human endothelial vascular cell line HECV.: percentage of wound edge distance after 24 h of treatment (T24) as compared to T0. G, H: representative images of the effects of skin peptides at 0.05 µg/ml vs controls (CTRL).
